# Supplementary material for: Epithelial cells release adenosine to promote local TNF production in response to polarity disruption
Source: Nat Commun. 2018 Nov 7;9:4675. doi: 10.1038/s41467-018-07114-z (PMC6220285; doi:10.1038/s41467-018-07114-z)
Supplement: Supplementary file 1 — Supplementary Information [file 41467_2018_7114_MOESM1_ESM.pdf]

Supplementary Information for

Epithelial cells release adenosine to promote local TNF production in response to polarity disruption

Poernbacher[1*] et al.

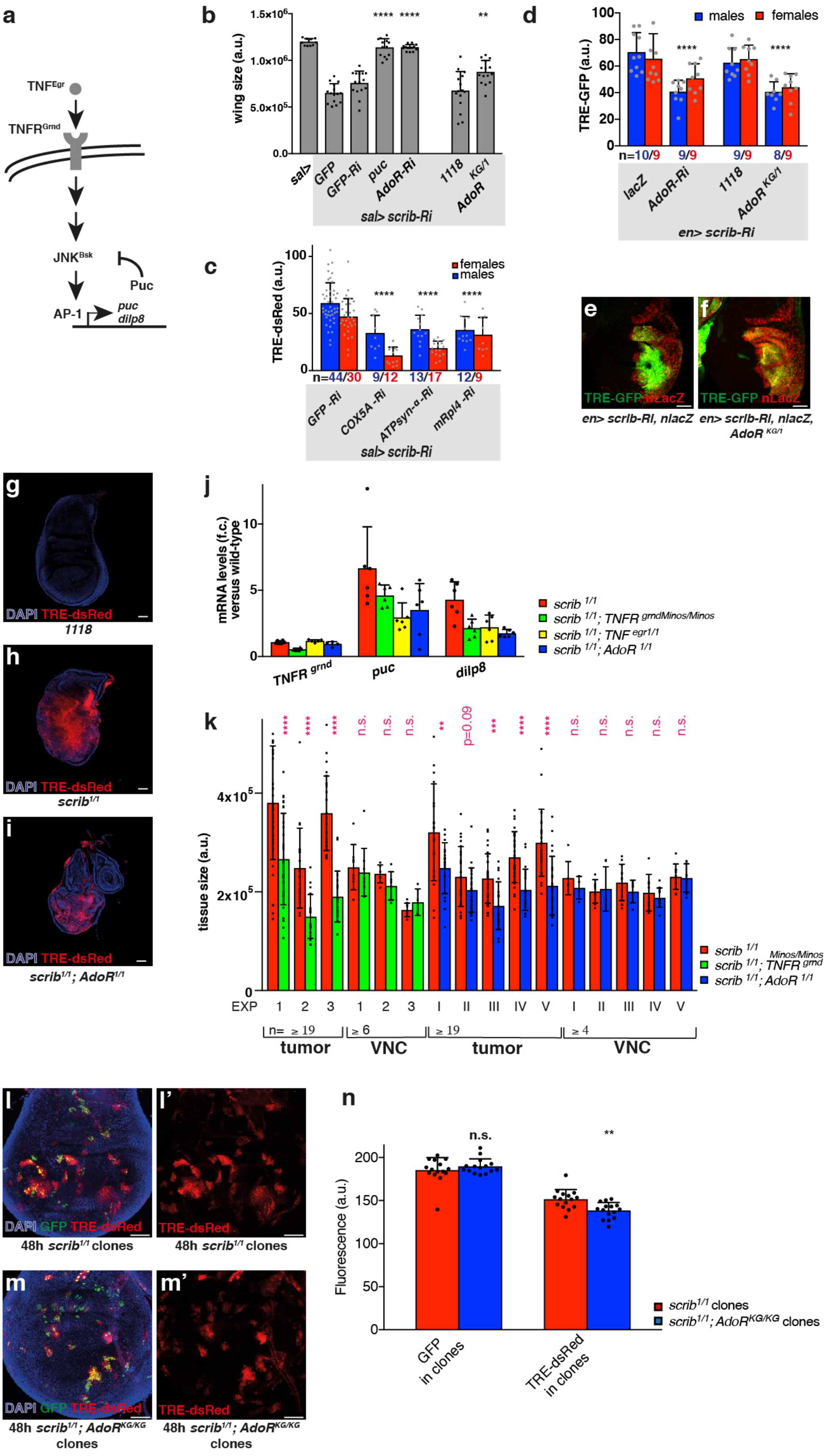
a
TNFEgr
TNFRGrnd
JNKBsk
Puc
AP-1
puc
dilp8
b
wing size (a.u.)
sal>
GFP
GFP-Ri
puc
AdoR-Ri
1118
AdoR KG/1
sal> scrib-Ri
c
TRE-dsRed (a.u.)
females
males
GFP -Ri
COX5A -Ri
ATPsyn-α-Ri
mRpl4 -Ri
sal> scrib-Ri
d
TRE-GFP (a.u.)
males
females
lacZ
AdoR-Ri
1118
AdoR KG/1
en> scrib-Ri
e
f
TRE-GFP nLacZ
en> scrib-RI, nlacZ
en> scrib-RI, nlacZ, AdoR KG/1
g
h
i
DAPI TRE-dsRed
1118
scrib1/1
scrib1/1; AdoR1/1
j
mRNA levels (f.c.) versus wild-type
TNFR grnd
puc
dilp8
scrib 1/1
scrib 1/1; TNFR grndMinos/Minos
scrib 1/1; TNF egr1/1
scrib 1/1; AdoR 1/1
k
tissue size (a.u.)
EXP
tumor
VNC
scrib 1/1
scrib 1/1; TNFR grnd Minos/Minos
scrib 1/1; AdoR 1/1
l
l'
DAPI GFP TRE-dsRed
TRE-dsRed
48h scrib1/1 clones
m
m'
48h scrib1/1; AdoRKG/KG clones
n
Fluorescence (a.u.)
GFP in clones
TRE-dsRed in clones
scrib1/1 clones
scrib1/1; AdoRKG/KG clones

Supplementary Fig. 1

AdoR signalling boosts JNK activation in *scrib* tumors but has only a very mild effect on *scrib* mutant clones.

(a) *Drosophila* TNF/JNK signalling.

(b) Surface area of wings obtained from male flies of the indicated genotypes. Reduced size correlates with increased melanisation and crumpling (n≥13).

(c) Knockdown of *ATP synthase*, *Cox5A* or *mRpL5* suppresses JNK signalling in *sal> scrib-Ri* discs.

(d-f) Depletion of *AdoR,* by RNAi or by mutation, reduces *TRE-GFP* activity in *en> scrib-Ri* discs.

(g-k) Wing discs from homozygous *scrib* mutant larvae activate *TRE-dsRed* (g, h) and upregulate the JNK target genes *puc* and *dilp8* (j). $TNFR^{grnd}$ expression, measured by RT-qPCR, is shown as a control. Fold changes are relative to *rp49*, n=6. Impairment of TNF^{Egr} or $TNFR^{Grnd}$ (TNF^{egr} *scrib* or $TNFR^{grnd}$ *scrib* double mutants) partially reduces JNK target gene expression (j) and tumor size (k). Here, the size of the ventral nerve cord (VNC) is used as an internal control (k). Similar effects are seen in *scrib AdoR* double mutant discs (i-k).

(l-n) *scrib* mutant clones activate *TRE-dsRed*. In *scrib AdoR* double mutant clones *TRE-dsRed* activity is slightly, albeit significantly, reduced. The graph in (n) represents quantifications of GFP and *TRE-dsRed* fluorescence from all the clones present in each of n=15 female wing discs for each genotype.

Scale bars, 50 μm. In graphs, means are shown, and error bars represent ±SD. $^{**}P < 0.01$, $^{***}P < 0.001$, $^{****}P < 0.0001$, unpaired two-tailed Student's t-test.

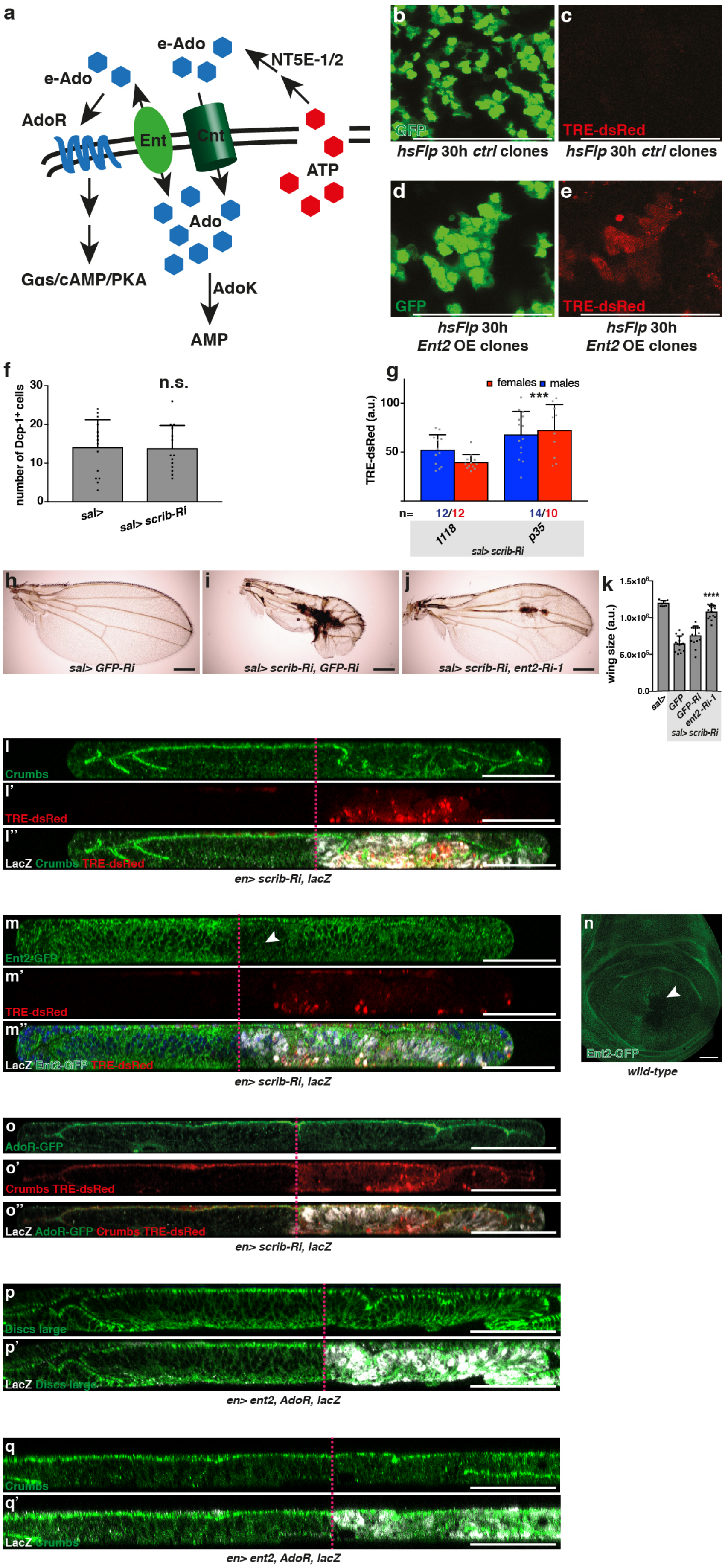
a
e-Ado
e-Ado
NT5E-1/2
AdoR
Ent
Cnt
ATP
Ado
Gαs/cAMP/PKA
AdoK
AMP
b
GFP
hsFlp 30h ctrl clones
c
TRE-dsRed
hsFlp 30h ctrl clones
d
GFP
hsFlp 30h Ent2 OE clones
e
TRE-dsRed
hsFlp 30h Ent2 OE clones
f
number of Dcp-1+ cells
n.s.
sal>
sal> scrib-Ri
g
TRE-dsRed (a.u.)
females
males
***
n= 12/12 14/10
1118
p35
sal> scrib-Ri
h
sal> GFP-Ri
i
sal> scrib-Ri, GFP-Ri
j
sal> scrib-Ri, ent2-Ri-1
k
wing size (a.u.)
****
sal>
GFP
GFP-Ri
ent2-Ri-1
sal> scrib-Ri
l
Crumbs
l'
TRE-dsRed
l''
LacZ Crumbs TRE-dsRed
en> scrib-Ri, lacZ
m
Ent2-GFP
m'
TRE-dsRed
m''
LacZ Ent2-GFP TRE-dsRed
en> scrib-Ri, lacZ
n
Ent2-GFP
wild-type
o
AdoR-GFP
o'
Crumbs TRE-dsRed
o''
LacZ AdoR-GFP Crumbs TRE-dsRed
en> scrib-Ri, lacZ
p
Discs large
p'
LacZ Discs large
en> ent2, AdoR, lacZ
q
Crumbs
q'
LacZ Crumbs
en> ent2, AdoR, lacZ

Supplementary Fig. 2

e-Ado signals in an autocrine manner and localisation of AdoR or Ent2 protein.

(a) Key regulators of e-Ado and purinergic signalling.

(b-e) Ent2-overexpressing clones activate *TRE-dsRed* cell-autonomously. Clones are marked by co-expression of GFP.

(f) Quantification of cells that are positive for the apoptosis marker Dcp-1 in the *sal*> domain of male wing discs of the indicated genotypes (n=14).

(g) Overexpression of p35, an inhibitor of apoptosis, does not reduce JNK signalling in *sal*> *scrib-Ri* discs.

(h-k) Knockdown of *ent2* partially rescues the defects of *sal*> *scrib-Ri* male wings (in k $n \geq 14$).

(l) *en*> *scrib Ri* discs have disrupted polarity in the posterior compartment (right side of pink line).

(m, o) *scrib* knockdown does not affect the levels or localization of Ent2-GFP expressed from a genomic fosmid (m) or of endogenously tagged AdoR-GFP (o). White arrowheads in (m) and (n) indicate a suspected position effect at the attP site (VK00033) where the fosmid was integrated. Both preparations are stained with anti-GFP antibodies.

(p, q) Overexpression of Ent2/AdoR in the posterior compartment (with *en*-Gal4) does not affect the distribution of epithelial polarity markers Discs large (p) and Crumbs (q).

Confocal images in (l-q) are cross-section xz views with posterior to the right and apical up. The A/P boundary is marked by a dotted pink line.

Scale bars, 50 µm (b-e, l-q) and 0.5 mm (h-j). In graphs means are shown, and error bars represent ±SD. **** $P < 0.0001$, *** $P < 0.001$, unpaired two-tailed Student's t-test. In (k) the effect of 'ent2-Ri' was compared to that of 'GFP-Ri' for statistical analysis.

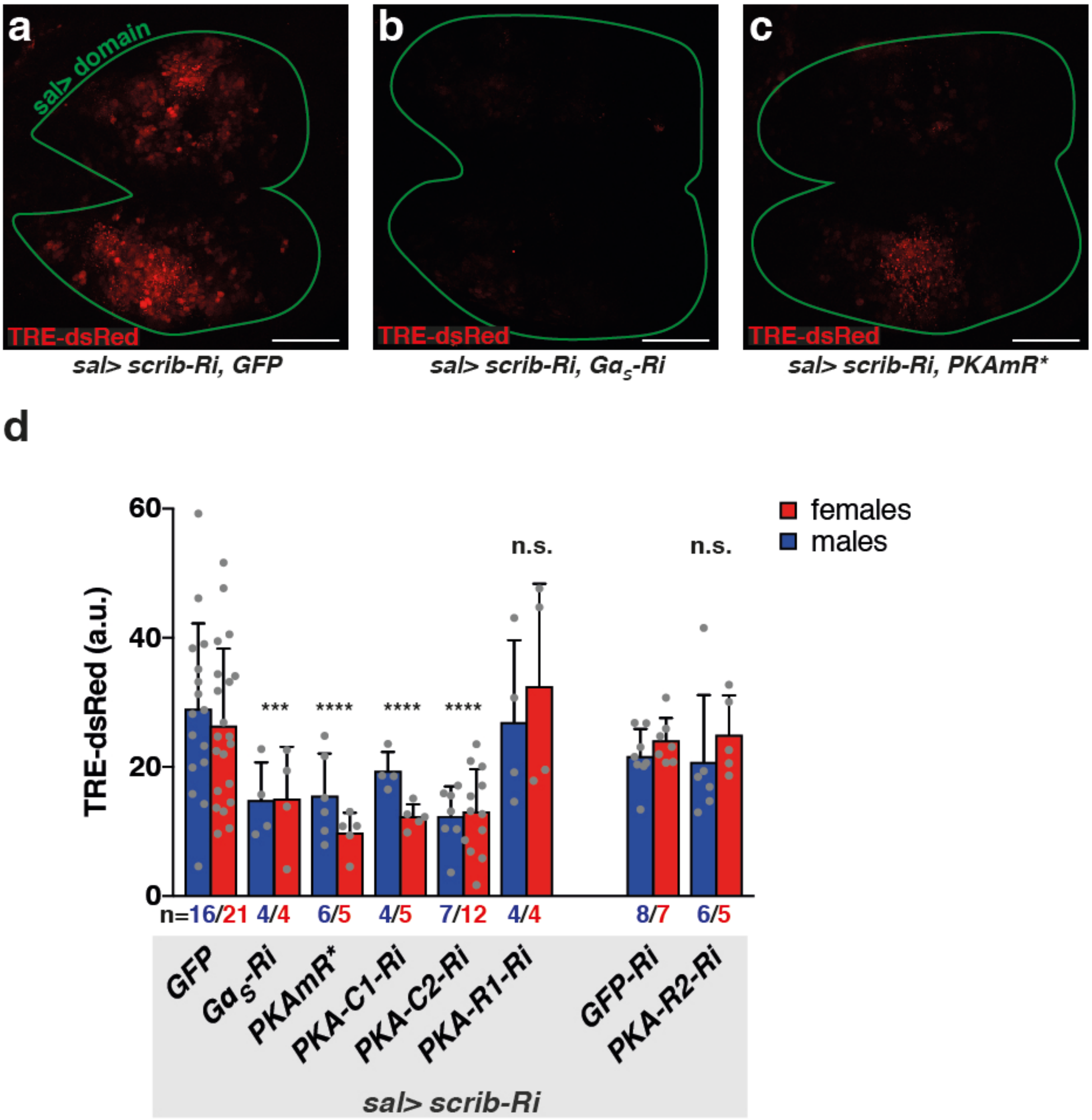

Supplementary Fig. 3

Gα_s/cAMP/PKA signalling is required for JNK activation in *scrib* deficient discs.

(a-d) *Gα_s-RNAi,* dominant negative PKA (*PKAmR**), or *PKA-C-RNAi* reduce *TRE-dsRed* expression in *sal> scrib-Ri* discs. Knockdown of *PKA-R1* or *PKA-R2*, which is expected to increase PKA activity, was used as a control.

*S*cale bars, 50 µm. In graphs, means are shown, and error bars represent ±SD; *** $P < 0.001$, ****$P < 0.0001$, unpaired two-tailed Student's t-test (males and females pooled together).

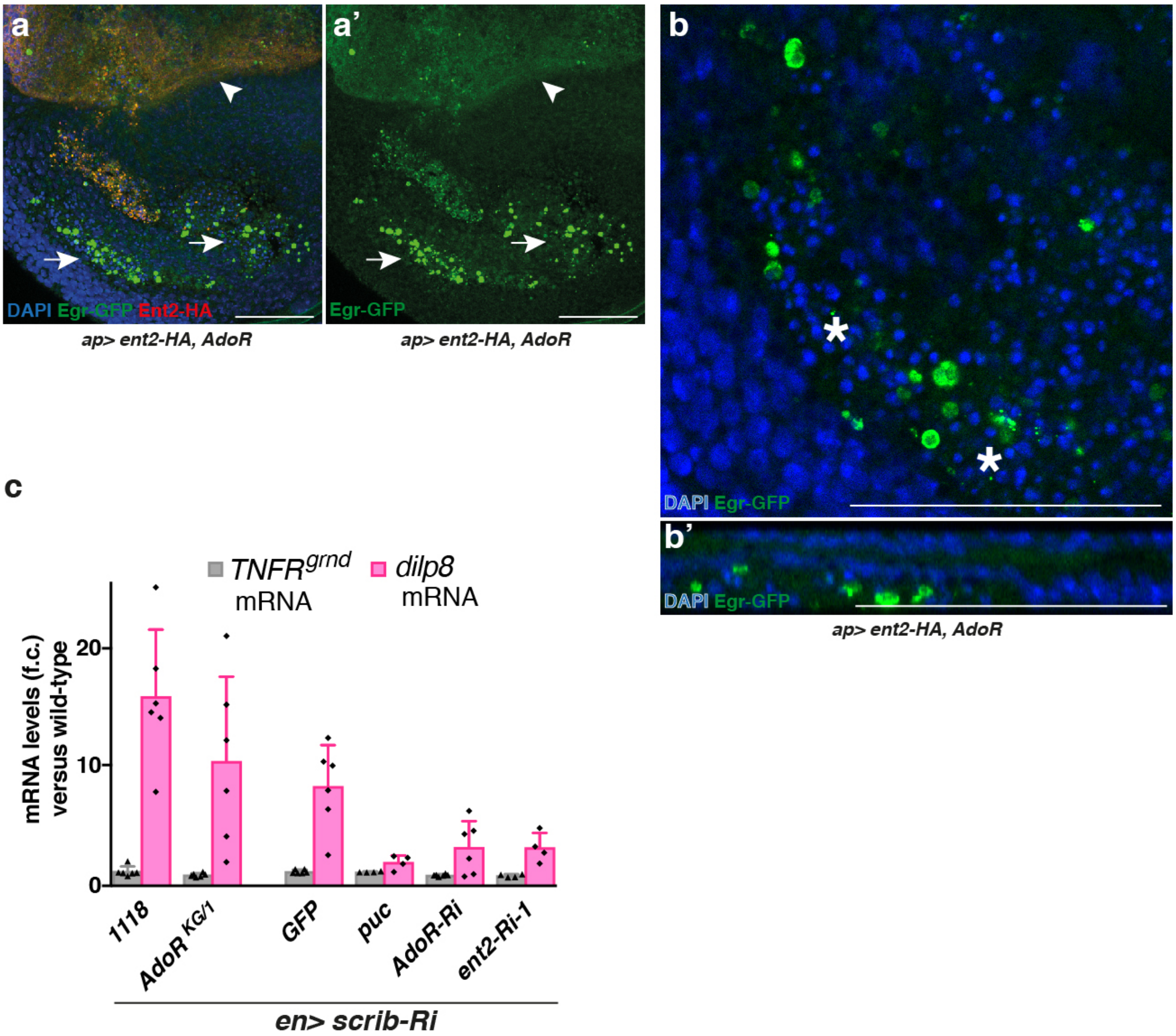

Supplementary Fig. 4

AdoR-mediated Egr expression in the disc epithelium does not arise from immune cells.

(a, b) As shown in Fig. 4c, overexpression of Ent2/AdoR with *sal*-Gal4 triggers expression of Egr-GFP within the epithelium without the involvement of recruited macrophages. Overexpression of Ent2/AdoR with the stronger *apterous*-Gal4 (*ap*-Gal4) also induces epithelial Egr-GFP expression (arrowheads in a). Because of the strong activation of JNK signalling, it additionally causes significant apoptosis, as evidenced by the presence of cellular debris, which are known to attract macrophages (asterisks in b). Therefore *ap-Gal4> ent2, AdoR* discs provide a situation where epithelial expression of TNF^{Egr} (arrowheads) and macrophage recruitment (arrows) can be seen, and distinguished, in the same preparation.

The distinct morphology of macrophages (small round cells that are Egr-GFP positive) can be seen at high magnification in panel b.

Scale bars are 50 μm (a) and 100 μm in (b).

(c) *en> scrib Ri* discs upregulate JNK target *dilp8* mRNA in an AdoR/Ent2-dependent and JNK-dependent fashion. $TNFR^{grnd}$ expression, measured by RT-qPCR, is shown as a control. Fold changes are relative to *rp49*, n≥4.
